# Sex Differences in the Incidence and Outcomes of Acute Myocardial Infarction in Spain, 2016–2018: A Matched-Pair Analysis

**DOI:** 10.3390/jcm10081795

**Published:** 2021-04-20

**Authors:** José M. de Miguel-Yanes, Rodrigo Jiménez-García, Valentin Hernandez-Barrera, Javier de Miguel-Díez, Nuria Muñoz-Rivas, Manuel Méndez-Bailón, Napoleón Pérez-Farinós, Marta López-Herranz, Ana Lopez-de-Andres

**Affiliations:** 1 Internal Medicine Department, Hospital General Universitario Gregorio Marañón, Instituto de Investigación Sanitaria Gregorio Marañón (IiSGM), Universidad Complutense de Madrid, 28040 Madrid, Spain; josemaria.demiguel@salud.madrid.org; 2 Department of Public Health & Maternal and Child Health, Faculty of Medicine, Universidad Complutense de Madrid, 28040 Madrid, Spain; anailo04@ucm.es; 3 Preventive Medicine and Public Health Teaching and Research Unit, Health Sciences Faculty, Universidad Rey Juan Carlos, Alcorcon, 28922 Madrid, Spain; valentin.hernandez@urjc.es; 4 Respiratory Care Department, Hospital General Universitario Gregorio Marañón, Instituto de Investigación Sanitaria Gregorio Marañón (IiSGM), Universidad Complutense de Madrid, 28040 Madrid, Spain; javier.miguel@salud.madrid.org; 5 Internal Medicine Department, Hospital Universitario Infanta Leonor, 28031 Madrid, Spain; nmunozr@salud.madrid.org; 6 Internal Medicine Department, Hospital Universitario Clínico San Carlos, Universidad Complutense de Madrid, 28040 Madrid, Spain; manuel.mendez@ucm.es; 7 Public Health and Psychiatry Department, Faculty of Medicine, Universidad de Malaga, 29010 Malaga, Spain; napoleon.perez@uma.es; 8 Nursing Department, Faculty of Nursing, Physiotherapy and Podology, Universidad Complutense de Madrid, 28040 Madrid, Spain; martal11@ucm.es

**Keywords:** myocardial infarction, gender, sex differences, STEMI, NSTEMI, in-hospital mortality

## Abstract

(1) Background: Our aim was to analyze the incidence, procedures, and in-hospital outcomes of myocardial infarction (MI) in Spain (2016–2018) according to sex. (2) Methods: We estimated the incidence of an ST elevation myocardial infarction (STEMI) and non-ST elevation myocardial infarction (NSTEMI) according to sex using the Spanish National Hospital Discharge Database. A matched-pair analysis was used. (3) Results: MI was coded in 156,826 patients aged ≥18 years (111,842 men and 44,984 women). Men showed higher incidence rates (205.0 vs. 77.8 per 100,000; *p* < 0.001; IRR = 2.81(95%CI:2.78–2.84)). After matching, the use of coronary artery by-pass grafting (CABG) (1.0% vs. 0.7%; *p* < 0.001) and percutaneous coronary intervention (PCI) (57.8% vs. 52.3%; *p* < 0.001) was higher among men with an STEMI, whereas the in-hospital mortality (IHM) remained higher among women (11.2% vs. 10.1%; *p* < 0.001). Likewise, CABG (1.9% vs. 3.3%; *p* < 0.001) and PCI (33.8% vs. 41.9%; *p* < 0.001) were less often used among women with an NSTEMI, but no sex-related differences were found in IHM. After adjusting for confounders, IHM was more than twofold higher for both men and women with an STEMI than those with an NSTEMI. Women with an STEMI had a 21% higher mortality risk than men (OR = 1.21(95%CI:1.13–1.29). (4) Conclusion: Men had higher incidence rates of MI than women. Women underwent invasive procedures less often and had a higher IHM when admitted for an STEMI.

## 1. Introduction

Cardiovascular disease is the leading cause of death globally [1]. There has been a decades-long debate on whether acute myocardial infarction (MI) is associated with a higher mortality risk in women as compared with men, especially at younger ages [2]. Indeed, some authors have described a sex–age interaction effect that could explain the worse outcomes initially seen for younger women after MI [3]. Subsequent research pointed to the fact that women received invasive therapies less often, mainly at advanced ages, and this distinct management seemed to account for the differences found in short-term mortality [4,5]. Very recently, Alkhouli M et al., using the Nationwide Inpatient Sample (NIS), analyzed sex-differences in the incidence and outcomes of ST elevation myocardial infarction (STEMI) and non-ST elevation myocardial infarction (NSTEMI), with data of a total of 6,720,639 weighted hospitalizations from 2001 to 2015 in the United States. The conclusions of this study were that the incidence of MI was lower among women, who also undergo invasive treatments less frequently regardless of their age. However, the negative impact of female sex on most outcomes is age-dependent, being more pronounced in young and middle-aged women [6]. In Spain, using hospital discharge data for the period 2005–2015, as in the USA, the incidence of STEMI and NSTEMI was higher among women, but the sex differences in in-hospital mortality (IHM) was dependent on the MI type, with women showing a lower IHM for NSTEMI and higher mortality for STEMI [7].

Nevertheless, research based on nationwide MI registries and administrative databases shows controversial results due to the different methodological approaches employed [6,7,8]. Most analyses adjust for the baseline characteristics of the patients, clinical variables, and use of invasive procedures using multivariable logistic regression models, but residual confounding often precludes reaching more definite conclusions [6,7,8]. Pair-matching enables a comparison of outcomes among non-uniformly-treated units to estimate the effect of the treatments while minimizing bias due to confounding caused by age and sex [9]. Another limitation found through comparing studies using information from discharge databases is that MI is defined using different versions of the International Classification of Disease (ICD) [6,7].

Here, we aimed to compare the incidence, clinical characteristics, use of therapeutic procedures, and in-hospital outcomes, namely, the length of hospital stay (LOHS) and in-hospital mortality (IHM), among women and men with a primary diagnosis of MI. We separately analyzed the patients with an STEMI and NSTEMI and compared the use of therapeutic procedures and in-hospital outcomes according to sex after matching by age, MI type, and year of hospitalization. Finally, variables associated with IHM were identified for both sexes and MI types.

## 2. Materials and Methods

### 2.1. Study Design and Data Source

The study design is observational and retrospective. We used the Spanish National Hospital Discharge Database (SNHDD), which includes up to 20 diagnoses and 20 procedures for each hospitalization coded with the ICD version 10 (ICD-10), as the data source. As this database is mandatory by law, over 95% of hospital discharges in Spanish hospitals are included. More detailed information on SNHDD is available online [10].

### 2.2. Study Population

Every hospital discharge, from 1 January 2016 to 3 December 2018, of a person aged 18 years or older, who had a primary diagnosis of MI, was included in our study population. ICD-10 codes for MI used in our investigation are shown in Supplementary Table S1. We had 17 hospitalizations with unknown/indeterminate sex values (≈0.01%) that were excluded for the purposes of this study. Those hospitalizations codified as “discharges against medical advice” in the variable of discharge type were very infrequent (*n* = 759; 0.48%), and these cases were considered as survivors at hospital discharge.

### 2.3. Study Variables

The study variables have been detailed in a previous investigation [11]. Briefly, we analyzed the incidence rates by age groups, IHM, LOHS, and the use revascularization procedures, such as coronary artery bypass grafting (CABG) and percutaneous coronary intervention (PCI), for men and women with an STEMI and NSTEMI. The algorisms described by Sundararajan et al. were applied to identify the conditions and calculate the Charlson Comorbidity Index (CCI) [12].

Information on cardiovascular risk factors and the use of invasive procedures or therapies was extracted with ICD 10 codes, which are shown in Supplementary Table S1.

We described and compared the proportion of men and women who had an “early discharge”, defined as an LOHS of under 72 h [13].

### 2.4. Matching Method

Given the differences in the age distribution, we conducted a pair-match analysis. We thus matched 22,184 of 23,594 (94.0%) women with an STEMI and 20,596 of 21,390 women with an NSTEMI (96.3%) with a man with an identical age, MI type, and year of hospitalization. Details on this method are available elsewhere [11].

### 2.5. Statistical Analysis

The descriptive statistics included absolute and relative frequencies (shown as percentages), means, medians, standard deviations, and interquartile ranges. For bivariate comparisons of men and women, chi-square, t-student, and Mann–Whitney tests were applied, as appropriate.

The multivariable analysis included Poisson and logistic regression, as described before [11,14]. Stata version 14 (College Station, TX, USA) was the statistical software used.

### 2.6. Ethical Aspects

According to the Spanish law, investigations using anonymized and public access [15] databases do not need approval by an ethics committee.

## 3. Results

In Spain, for the years from 2016 to 2018, the SNHDD included 156,826 patients aged 18 years or over with a primary diagnosis of MI. Men represented 71.3% (111,842), and women represented 28.7% (44,984).

### 3.1. Incidence of STEMI and NSTEMI According to Sex

Table 1 shows the incidence rates according to the MI type, sex, and age groups. For both sexes, the proportion of patients with an STEMI was higher than that of patients with an NSTEMI. Overall, the incidence rate of MI was higher among men than among women (205.0 vs. 77.8 per 100,000; *p* < 0.001), resulting in an adjusted IRR = 2.81 (95%CI 2.78–2.84). For STEMI, we obtained an IRR = 3.14 (95%CI 3.10–3.18), and for NSTEMI, IRR = 2.51(95%CI 2.46–2.56), for men vs. women. Men had higher incidence rates of STEMI and NSTEMI than women in all of the age groups analyzed. In either sex, the incidence of both types of MI increased with age, reaching the highest incidence among those aged ≥75 years.

### 3.2. Clinical Characteristics, Therapeutic Procedures, and Hospital Outcomes for Men and Women with an STEMI and NSTEMI

The clinical characteristics, therapeutic procedures, and hospital outcomes, before and after matching for men and women who suffered an STEMI, are shown in Table 2 and Supplementary Table S2. We found significant differences in the distribution of the ICD 10 codes for STEMI between sexes (Table 2): there was a higher incidence of STEMI involving well-defined territories (e.g., “left anterior descending coronary artery” or “right coronary artery”) among men, although there was a higher proportion of “unspecified site” STEMI among women (17.7% vs.11.4%; *p* < 0.001).

Before matching, women were significantly older and had a higher mean CCI and a prevalence of obesity, hypertension, atrial fibrillation, and most conditions were included in the CCI. Men were more frequently coded for previous MI, peripheral vascular disease, COPD, liver disease, and cancer (Table 2 and Supplementary Table S2). Regarding therapeutic procedures, thrombolytic therapy, CABG, and PCI were more commonly used in men than in women. Early discharge was more frequent among men than women, prior to matching (18.5% vs. 14.8%; *p* < 0.001) and after matching (16.7% vs. 15.2%; *p* < 0.001). The crude LOHS and IHM showed significantly worse figures among women. Once matching was conducted, the use of CABG (1.0% vs. 0.7%; *p* < 0.001) and PCI (57.8% vs. 52.3%; *p* < 0.001) was higher among men, whereas cardiogenic shock (6.8% vs. 5.7%; *p* < 0.001) and IHM (11.2% vs. 10.1%; *p* < 0.001) remained higher among women.

Table 3 and Supplementary Table S3 show a comparison of men and women admitted for an NSTEMI. As seen for STEMI, women had a significantly higher prevalence of most clinical conditions, with the same exceptions reported for STEMI. Before matching, women had a lower proportion of early discharge, they less frequently had a code for CABG and PCI, and they showed a higher IHM and LOHS than men. After matching, CABG (1.9% vs. 3.3%; *p* < 0.001) and PCI (33.8% vs. 41.9%; *p* < 0.001) were less often used among women, although the differences in early discharge, IHM, and LOHS were no longer statistically significant.

### 3.3. Variables Associated with IHM after Multivariable Analysis

The results of the multivariable logistic regression analyses are shown in Table 4. In the five models constructed, the risk of dying in the hospital increased with age and with the presence of congestive heart failure, cerebrovascular disease, or dementia. Renal disease and atrial fibrillation increased the risk of IHM only among women. Obesity was associated with a lower IHM in all the models. The use of CABG or PCI was associated with a lower IHM, while mechanical ventilation was associated with a higher IHM.

After adjusting for possible confounders, the risk of dying during the hospital stay was more than twofold higher for men admitted for an STEMI, as compared with that for men admitted for an NSTEMI (OR = 2.26; 95%CI 2.08–2.45). Among women, this risk was even higher (OR = 2.76; 95%CI 2.56–2.98). In the model used, including men and women with an STEMI, OR = 1.21 (95%CI 1.13–1.29), women had a 21% higher mortality risk. However, no significant differences according to sex were found after multivariable adjustment for people admitted for an NSTEMI.

## 4. Discussion

Here, we found that men had higher incidence rates of STEMI and NSTEMI than women in all of the age groups analyzed. After pair-matching according to age, MI code, and year of hospitalization, the use of CABG and PCI was lower in women. Proceeding with CABG or PCI appeared to be associated with a lower IHM. IHM was significantly higher in women admitted to the hospital for an STEMI. IHM was more than twofold higher when both men and women were admitted for an STEMI, compared to those admitted for an NSTEMI. In the fully-adjusted model, women admitted to the hospital for an STEMI had a 21% higher adjusted risk of dying than men, but no differences were found in the case of admission for NSTEMI.

According to our database, men had higher incidence rates of STEMI and NSTEMI than women, and the incidence increased with age. These trends are in accordance with what has been previously described by many authors [6,16,17]. While the American Heart Association has summarized the particularities of MI in women in a document, showing the increasing weight of cardiovascular mortality in women [18], virtually every study shows persistently higher MI incidence rates in men. What is striking is that these higher rates have been found to be incompletely explained by the established risk factors [19].

During admission for STEMI, we found that the female sex was associated with a higher IHM, and the higher mortality risk among women remained in the multivariable regression model. The reason for this is probably multifactorial. Before matching, but also once matching was completed, we could see that the use of CABG and PCI was higher among men, despite the fact that cardiogenic shock was more often coded in women. This finding has been previously reported by others. Alkhouli M et al. reported that women received a less-invasive treatment of both STEMI and NSTEMI across all age groups [6]. This is a relevant issue, since women may be more vulnerable than men to long-standing untreated ischemia [20]. The reason for the lower use of coronary revascularization in women is unclear, since the use of CABG or PCI has been associated with a lower IHM in previous research works [16,21]. This less invasive pattern in women has been formerly discussed by other investigators [6,16,21,22]. Older studies speculated that women are less likely to receive timely in-hospital reperfusion therapy due to prehospital delays in hospital presentation [19], but the influence of this circumstance on PCI or CABG should be lower than on pharmacologic thrombolysis. Despite the more than probable influence of the lower rates of CABG and PCI on the higher mortality shown for women, we must notice that higher mortality rates have been reported for women who undergo PCI as well [23,24]. In any case, despite what other authors have reported [25], in our study, the mortality gap between sexes was apparently not explained by age or comorbidities. Previous studies have reported an interaction between age, sex, and outcomes of MI [6,16,19] A recent report with almost 7 million MI patients observed that younger women have generally worse outcomes, but older women have better outcomes than their male counterparts. Specifically, the risk of dying in the hospital was higher among women than men for those aged <65 following an NSTEMI and those aged <85 following an STEMI [6]. In our investigation, only STEMI women had a significantly higher IHM, after matching and logistic regression adjustment. In our opinion, this lack of association for NSTEMI may be a consequence of the interaction with age reported by other authors [6,19]. Furthermore, a previous report conducted in Spain from 2005 to 2015, using similar methods to ours, reported that women exhibited a higher mortality for STEMI and lower mortality for NSTEMI [7]. More research is needed to clarify this issue in our country.

The sex differences in the pathophysiology underlying coronary disease, described in observational studies, could partly explain the worse outcomes among women with MI [26]. Women have more coronary microvascular dysfunctions and coronary flow reserve impairments than men [26]. These two conditions can result in MI with non-obstructive coronary arteries (MINOCA) [27]. MINOCA is much more frequent, by almost five times, among women than men, representing up to 10% for STEMI and 15% for NSTEMI among women [26,27,28]. Other pathophysiology mechanisms that also contribute to MINOCA that are more frequent among women include spontaneous coronary artery dissection and plaque erosion [26]. MINOCA patients have significantly worse outcomes, compared to age-matched controls [29].

During admission for NSTEMI, invasive procedures were less often used among women too, although the differences in IHM were no longer statistically significant. This may indicate that other factors may be responsible for the different sex-associated mortality rates seen in people admitted for an STEMI. Moreover, this raises the issue that the final cause of death in people admitted for an NSTEMI could be something other than cardiovascular, but the evidence points to the contrary [30,31].

Obesity was associated with a lower IHM in all the logistic regression models in our study, which is consistent with some previous research [32,33], but this finding has long been controversial. The reasons why obesity could be associated with a lower mortality risk after MI is not fully understood, but factors such as wider coronary artery diameters in obese patients [34] and recent pathophysiologic insights that suggest that excessive energy stores might confer some benefit in the context of disease related catabolic conditions [35] have been proposed.

The risk of dying during the hospital stay was more than twofold higher for men and women admitted for an STEMI, as compared with men and women admitted for an NSTEMI. This used to be the rule in older studies [36], but more recent reports show only slightly worse outcomes for STEMI [37] or even similar IHM rates for both conditions [38]. A more invasive management of NSTEMI, which shares the presence of myocardial necrosis with the STEMI, probably explains the similar rates found in more recent research work [37,38].

The higher proportion of *“unspecified site”* STEMI in women than in men found in our population, when ICD 10 codes are used, could be explained in several ways. First, the lower utilization rate of invasive procedures. Second, the more complex interpretation of the electrocardiogram findings or the higher presence of left-bundle branch block among women. Third, as commented before, the higher proportion of myocardial infarction with non-obstructed coronary arteries among women [26].

Walli-Attaei et al. have suggested that the lower number of revascularization procedures observed in women might be partly explained by the lower burden of atherosclerosis in women. Therefore, the likelihood of the treating physicians ordering invasive procedures to evaluate macrovascular coronary disease in women may be decreased, thus leading to a less accurate diagnosis [16].

### Limitations

Our study has a number of limitations that must be considered. The data source used is an administrative database, created primarily for billing purposes, which is supported by the information that physicians record in the discharge report, and this also depends on manual coding by the administrative staff. Therefore, the risk of under-coding, over-coding, or erroneous coding cannot be ruled out.

As described for other administrative databases, the limited information available in the SNHDD means that we lack findings regarding electrocardiography and angiography, access sites, the characteristics of the MI culprit artery, perioperative medications, and the reasons for invasive management strategies [6].

In addition, anonymity precludes the extraction of some specific pieces of information (e.g., people who moved from one hospital to another would appear twice), and patients who had multiple events during the observation time interval may have been included more than once in the study. Outcomes after the patients are discharged from the hospital are not available.

We have only included patients with a primary diagnosis of MI, so those who suffered this event during the hospitalization are not analyzed. However, if MI in any diagnosis code was included, it would have been impossible to determine if IHM was a consequence of the MI or not [6].

As mentioned in the methods section, a small proportion of patients had unknown/indeterminate sex values (≈0.01%) and were excluded for the purposes of this study or were classified as “discharges against medical advice” (0.48%) and were included in the analysis. In our opinion, the possible effect of these small percentages in our results is most likely irrelevant.

Finally, despite a pair-matching process that contributed to the attenuation of sex-related differences in baseline characteristics and clinical variables, a complete elimination of residual confounding is not possible in observational studies.

In addition to these limitations, the strength of our findings lies in the large sample size, with data from over 156,826 episodes of MI, the widespread coverage of the population of an entire country (>95% of all hospital admissions), the standardized methodology, which has been extensively used in research in Spain [7,11,14], and the good reliability of acute coronary syndrome coding in the SNHDD [39].

## 5. Conclusions

In Spain, the analysis of national representative hospital discharge data for recent years showed that men had higher incidence rates of MI (STEMI and NSTEMI) than women. Sex differences exist, as women less often underwent invasive procedures, irrespective of the MI type, and had a higher IHM when admitted for an STEMI, after adjusting for confounding variables. Future research should focus on eliminating these sex-related disparities in our health system.

## Figures and Tables

**Table 1:** Incidence rates of hospital admission with a primary diagnosis of myocardial infarction, with and without ST segment elevation, according to sex and age group in Spain (2016–18).

		Men	Women	
Myocardial Infarction Type	Age Groups	*n* (Inc/10^5^)	*n* (Inc/10^5^)	*p*-Value
STEMI	18–44 years	4375(18.3)	730(3.1)	<0.001
	45–59 years	23,197(149.9)	4174(26.4)	<0.001
	60–74 years	22,863(223.8)	6707(61.7)	<0.001
	≥75 years	14,278(287.6)	11,983(159.8)	<0.001
	All age groups	64,713(118.6)	23,594(40.8)	<0.001
NSTEMI	18–44 years	1608(6.7)	390(1.7)	<0.001
	45–59 years	11,399(73.7)	2572(16.2)	<0.001
	60–74 years	17,652(172.8)	5912(54.4)	<0.001
	≥75 years	16,470(331.8)	12,516(166.9)	<0.001
	All age groups	47,129(86.4)	21,390(37.0)	<0.001
Total	18–44 years	5983(25.0)	1120(4.7)	<0.001
	45–59 years	34,596(223.6)	6746(42.6)	<0.001
	60–74 years	40,515(396.6)	12,619(116.0)	<0.001
	≥75 years	30,748(619.4)	24,499(326.7)	<0.001
	All age groups	111,842(205.0)	44,984(77.8)	<0.001

STEMI: ST-elevation myocardial infarction. NSTEMI: Non-ST elevation myocardial infarction. Inc/10^5^: Incidence per 100,000 population.

**Table 2:** Clinical characteristics and hospital outcomes, before and after matching by age and myocardial infarction type (ICD-10), for men and women suffering from an STEMI.

Variable	Before Matching			After Matching		
	Men	Women	*p*-Value	Men	Women	*p*-Value
STEMI involving left main coronary artery, *n* (%)	375 (0.6)	138 (0.6)	<0.001	112 (0.5)	112 (0.5)	MV
STEMI involving left anterior descending coronary artery, *n* (%)	8658 (13.4)	2732 (11.6)	<0.001	2700 (12.2)	2700 (12.2)	MV
STEMI involving other coronary artery of anterior wall, *n* (%)	15,301 (23.6)	5854 (24.8)	<0.001	5501 (24.8)	5501 (24.8)	MV
STEMI involving right coronary artery, *n* (%)	9799 (15.1)	2974 (12.6)	<0.001	2881 (13.0)	2881 (13.0)	MV
STEMI involving other coronary artery of inferior wall, *n* (%)	18,061 (27.9)	5876 (24.9)	<0.001	5550 (25.0)	5550 (25.0)	MV
STEMI involving left circumflex coronary artery, *n* (%)	1516 (2.3)	372 (1.6)	<0.001	360 (1.6)	360 (1.6)	MV
STEMI involving other sites, *n* (%)	3615 (5.6)	1480 (6.3)	<0.001	1380 (6.2)	1380 (6.2)	MV
STEMI of unspecified site, *n* (%)	7388 (11.4)	4168 (17.7)	<0.001	3700 (16.7)	3700 (16.7)	MV
Age, mean (SD)	63.3 (13.2)	72.6 (14.2)	<0.001	71.4 (13.7)	71.4 (13.7)	MV
CCI, mean (SD)	0.6 (0.9)	0.8 (1.0)	<0.001	0.8 (1.0)	0.8 (1.0)	0.001
CCI 0, *n* (%)	37,747 (58.3)	11,190 (47.4)	<0.001	10,850 (48.9)	10,742 (48.4)	0.001
CCI 1, *n* (%)	17,485 (27.0)	7414 (31.4)	<0.001	6599 (29.8)	6935 (31.2)	0.001
CCI 2, *n* (%)	6427 (9.9)	3471 (14.7)	<0.001	3020 (13.6)	3147 (14.2)	0.001
CCI ≥3, *n* (%)	3054 (4.7)	1519 (6.4)	<0.001	1715 (7.7)	1360 (6.1)	0.001
Obesity, *n* (%)	7585 (11.7)	3130 (13.3)	<0.001	2026 (9.1)	3048 (13.7)	<0.001
Hypertension, *n* (%)	27,677 (42.8)	11,993 (50.8)	<0.001	10,031 (45.2)	11,319 (51.0)	<0.001
Lipid metabolism disorders, *n* (%)	28,775 (44.5)	10,608 (45.0)	0.190	9531 (43.0)	10,105 (45.5)	<0.001
Atrial fibrillation, *n* (%)	6425 (9.9)	3904 (16.6)	<0.001	3264 (14.7)	3513 (15.8)	0.001
Cardiogenic shock, *n* (%)	3098 (4.8)	1600 (6.8)	<0.001	1266 (5.7)	1506 (6.8)	<0.001
Previous infarction, *n* (%)	4700 (7.3)	1134 (4.8)	<0.001	1876 (8.5)	1046 (4.7)	<0.001
Thrombolytic therapy, *n* (%)	3263 (5.0)	959 (4.1)	<0.001	930 (4.2)	935 (4.2)	0.906
Vasopressor medication, *n* (%)	763 (1.2)	323 (1.4)	0.153	288 (1.3)	306 (1.4)	0.457
Mechanical ventilation, *n* (%)	4010 (6.2)	1445 (6.1)	0.694	1460 (6.6)	1407 (6.3)	0.306
CABG, *n* (%)	719 (1.1)	145 (0.6)	<0.001	223 (1.0)	145 (0.7)	<0.001
PCI, *n* (%)	41,675 (64.4)	11,848 (50.2)	<0.001	12,816 (57.8)	11,596 (52.3)	<0.001
Early discharge, *n* (%)	11,994 (18.5)	3486 (14.8)	<0.001	3703 (16.7)	3363 (15.2)	<0.001
LOHS, median (IQR)	5 (4)	6 (5)	<0.001	5 (5)	5 (5)	0.077
In-hospital mortality, *n* (%)	4032 (6.2)	3050 (12.9)	<0.001	2246 (10.1)	2619 (11.2)	<0.001

ICD-10: International Classification of Disease version 10. STEMI: ST elevation myocardial infarction. CCI: Charlson comorbidity index. CABG: Coronary artery bypass grafting. PCI: Percutaneous coronary intervention. LOHS: Length of hospital stay. MV: Matching variable.

**Table 3:** Clinical characteristics and hospital outcomes, before and after matching by age, for men and women suffering from an NSTEMI.

	Without Matching			After Matching		
	Men	Women	*p*-Value	Men	Women	*p*-Value
NSTEMI, *n* (%)	47,129 (100)	21,390 (100)	NA	20,596 (100)	20,596 (100)	MV
Age, mean (SD)	68.1 (13.0)	75.0 (12.7)	<0.001	74.3 (12.5)	74.3 (12.5)	MV
CCI, mean (SD)	1.0 (1.1)	1.0 (1.1)	<0.001	1.1 (1.2)	1.0 (1.1)	<0.001
CCI 0, *n* (%)	21,450 (45.5)	8618 (40.3)	<0.001	7838 (38.1)	8418 (40.9)	<0.001
CCI 1, *n* (%)	13,510 (28.7)	6650 (31.1)	<0.001	6230 (30.3)	6365 (30.9)	<0.001
CCI 2, *n* (%)	7193 (15.3)	3971 (18.6)	<0.001	3766 (18.3)	3772 (18.3)	<0.001
CCI ≥3, *n* (%)	4976 (10.6)	2151 (10.1)	<0.001	2762 (13.4)	2041 (9.9)	<0.001
Obesity, *n* (%)	6142 (13.0)	3362 (15.7)	<0.001	2162 (10.5)	3329 (16.2)	<0.001
Hypertension, *n* (%)	23,144 (49.1)	11,332 (53.0)	<0.001	10,119 (49.1)	10,982 (53.3)	<0.001
Lipid metabolism disorders, *n* (%)	24,376 (51.7)	10,776 (50.4)	<0.001	10,364 (50.3)	10,522 (51.1)	0.119
Atrial fibrillation, *n* (%)	6567 (13.9)	4231 (19.8)	<0.001	3799 (18.5)	3980 (19.3)	0.023
Cardiogenic shock, *n* (%)	750 (1.6)	391 (1.8)	0.03	410 (2.0)	378 (1.8)	0.250
Previous infarction, *n* (%)	6819 (14.5)	2289 (10.7)	<0.001	3241 (15.7)	2196 (10.7)	<0.001
Thrombolytic therapy, *n* (%)	420 (0.9)	170 (0.8)	0.21	172 (0.8)	167 (0.8)	0.785
Vasopressor medication, *n* (%)	301 (0.6)	143 (0.7)	0.65	138 (0.7)	141 (0.7)	0.857
Mechanical ventilation, *n* (%)	1775 (3.8)	787 (3.7)	0.58	801 (3.9)	777 (3.8)	0.538
CABG, *n* (%)	1810 (3.8)	387 (1.8)	<0.001	678 (3.3)	386 (1.9)	<0.001
PCI, *n* (%)	21,809 (46.3)	7033 (33.0)	<0.001	8626 (41.9)	6965 (33.8)	<0.001
Early discharge, *n* (%)	7253 (15.4)	2617 (12.2)	<0.001	2645 (12.8)	2546 (12.4)	<0.001
LOHS, median (IQR)	5 (6)	6 (5)	<0.001	6 (5)	6 (5)	0.097
In-hospital mortality, *n* (%)	1909 (4.1)	1420 (6.6)	<0.001	1264 (6.1)	1281 (6.2)	0.728

ICD-10: International Classification of Disease version 10. NSTEMI: Non-ST elevation myocardial infarction. CCI: Charlson comorbidity index. CABG: Coronary artery bypass grafting. PCI: Percutaneous coronary intervention. LOHS: Length of hospital stay. MV: Matching variable. NA: Not Applicable.

**Table 4:** Multivariable logistic regression to identify variables independently associated with in-hospital mortality among men and women with an STEMI or NSTEMI and for both sexes according to myocardial infarction type.

Variable	Men	Women	STEMI	NSTEMI	Both Sexes, STEMI Plus NSTEMI
	OR (95%CI)	OR (95%CI)	OR (95%CI)	OR (95%CI)	OR (95%CI)
18–44 years	1	1	1	1	1
45–59 years	0.99 (0.57–1.71)	1.55 (0.94–2.55)	1.24 (0.84–1.83)	1.89 (0.58–6.17)	1.28 (0.89–1.85)
60–74 years	2.55 (1.52–4.27)	3.22 (2–5.19)	2.77 (1.91–4.02)	4.99 (1.59–15.66)	2.95 (2.07–4.18)
≥75 years	6.81 (4.08–11.36)	9.04 (5.63–14.5)	8.06 (5.58–11.66)	12.35 (3.95–38.6)	8.07 (5.70–11.42)
Obesity	0.73 (0.62–0.86)	0.77 (0.68–0.87)	0.75 (0.66–0.85)	0.76 (0.65–0.89)	0.75 (0.68–0.83)
Chronic renal disease	NS	1.18 (1.04–1.34)	NS	NS	1.1 (1.01–1.20)
Atrial fibrillation	NS	1.15 (1.06–1.25)	NS	1.21 (1.10–1.34)	1.1 (1.03–1.17)
Congestive heart failure	1.55 (1.39–1.72)	1.38 (1.23–1.53)	1.26 (1.14–1.39)	1.77 (1.57–2.01)	1.45 (1.34–1.56)
Cerebrovascular disease	1.13 (1.01–1.27)	1.49 (1.28–1.74)	1.38 (1.20–1.60)	1.2 (1.01–1.43)	1.31 (1.17–1.47)
Dementia	1.86 (1.54–2.23)	2.06 (1.78–2.39)	1.82 (1.57–2.11)	2.16 (1.81–2.58)	1.95 (1.74–2.19)
Mechanical ventilation	10.5 (9.43–11.7)	8.55 (7.68–9.52)	10.68 (9.72–11.74)	7.35 (6.45–8.38)	9.47 (8.78–10.22)
CABG	0.68 (0.51–0.89)	0.56 (0.39–0.81)	0.59 (0.41–0.85)	0.7 (0.53–0.93)	0.63 (0.51–0.79)
PCI	0.35 (0.32–0.38)	0.36 (0.33–0.39)	0.37 (0.34–0.40)	0.3 (0.27–0.35)	0.35 (0.33–0.38)
STEMI	2.26 (2.08–2.45)	2.76 (2.56–2.98)	NA	NA	2.51 (2.37–2.65)
Women	NA	NA	1.21 (1.13–1.29)	NS	1.11 (1.05–1.17)

STEMI: ST elevation myocardial infarction. NSTEMI: Non-ST elevation myocardial infarction. OR (95%CI): Odds Ratio (95% confidence interval). CABG: Coronary artery bypass grafting. PCI: Percutaneous coronary intervention. NA: Not applicable. NS: Not significant.

## Data Availability

The conditions imposed by the Spanish Ministry of Health to provide us with the SHHDD include that we cannot share the databases with any other investigator, and the files from the databases must be deleted once the analysis has been concluded [10]. Therefore, it is forbidden to upload the databases in any public repository.

## Supplementary Materials

The following are available online at https://www.mdpi.com/article/10.3390/jcm10081795/s1: Table S1: International Classification of Disease 10th edition (ICD-10)—codes for the clinical diagnoses and procedures used in this investigation; Table S2: Prevalence of conditions included in the Charlson comorbidity index (CCI), before and after matching by age and myocardial infarction type (ICD-10), for men and women suffering from a STEMI. Table S3: Prevalence of conditions included in the Charlson Comorbidity Index (CCI) before and after matching by age for men and women suffering a NSTEMI.
