# Supplementary material for: Sex Differences in the Incidence and Outcomes of Acute Myocardial Infarction in Spain, 2016–2018: A Matched-Pair Analysis
Source: J Clin Med. 2021 Apr 20;10(8):1795. doi: 10.3390/jcm10081795 (PMC8074598; doi:10.3390/jcm10081795)
Supplement: Supplementary file 1 [file jcm-10-01795-s001.zip › jcm-1127804-supplementary.pdf]

# Supplementary Material

**Supplementary Table S1**. International Classification of Disease 10th edition (ICD-10) codes for the clinical diagnoses and procedures used in this investigation.

| Clinical diagnoses and procedures | ICD-10 codes |
|---|---|
| STEMI involving left main coronary artery | I21.01 |
| STEMI involving left anterior descending coronary artery | I21.02 |
| STEMI involving other coronary artery of anterior wall | I21.09 |
| STEMI involving right coronary artery | I21.11 |
| STEMI involving other coronary artery of inferior wall | I21.19 |
| STEMI involving left circumflex coronary artery | I21.21 |
| STEMI involving other sites | I21.29 |
| STEMI of unspecified site | I21.3 |
| NSTEMI | I21.4 |
| Obesity | E66.X |
| Hypertension | I10, I16.6 |
| Lipid metabolism disorders | E78.0X-E78.5 |
| Atrial fibrillation | I48.0, I48.1, I48.2, I48.91 |
| Cardiogenic shock | R57.0 |
| Previous myocardial infarction | I25.2 |
| Mechanical ventilation | 5A1945Z, 5A1955Z, 5A1935Z,5A09357, 5A09457, 5A09557 |
| Thrombolytic therapy | 3E03317, 3E04317, 3E05317, 3E06317, 3E08317 |
| Vasopressor medication | 3E030XZ, 3E033XZ, 3E040XZ, 3E043XZ, 3E050XZ, 3E053XZ, 3E060XZ, 3E063XZ |
| CABG | 02100XX, 02110XX, 02120XX, 02130XX |
| PCI | 02703XX, 02713XX, 02723XX, 02733XX |

STEMI: ST elevation myocardial infarction. NSTEMI: non-ST elevation myocardial infarction. CABG: Coronary artery bypass grafting. PCI: Percutaneous coronary intervention.

**Supplementary Table S2**. Prevalence of conditions included in the Charlson comorbidity index (CCI) before and after matching by age and myocardial infarction type (ICD-10), for men and women suffering a STEMI.

| | BEFORE MATCHING | | | AFTER MATCHING | | |
|---|---|---|---|---|---|---|
| | **Men** | **Women** | **p-value** | **Men** | **Women** | **p-value** |
| Congestive heart failure, n(%) | 8,217(12.7) | 4,870(20.6) | <0.001 | 3,725(16.8) | 4,320(19.5) | <0.001 |
| Peripheral vascular disease, n(%) | 3,072(4.8) | 735(3.1) | <0.001 | 1,352(6.1) | 696(3.1) | <0.001 |
| Cerebrovascular disease, n(%) | 1,591(2.5) | 914(3.9) | <0.001 | 771(3.5) | 837(3.8) | 0.094 |
| Dementia, n(%) | 595(0.9) | 846(3.6) | <0.001 | 437(2.0) | 718(3.2) | <0.001 |
| COPD, n(%) | 5,240(8.1) | 1,748(7.4) | 0.001 | 2,514(11.3) | 1,654(7.5) | <0.001 |
| Rheumatoid disease, n(%) | 526(0.8) | 548(2.3) | <0.001 | 264(1.2) | 515(2.3) | <0.001 |
| Peptic ulcer, n(%) | 237(0.4) | 55(0.2) | 0.002 | 107(0.5) | 52(0.2) | <0.001 |
| Mild and moderate/severe liver disease, n(%) | 1,514(2.3) | 426(1.8) | <0.001 | 481(2.2) | 412(1.9) | 0.020 |
| Diabetes, n(%) | 15,606(24.1) | 7,027(29.8) | <0.001 | 6,104(27.5) | 6,634(29.9) | <0.001 |
| Hemiplegia/Paraplegia, n(%) | 204(0.3) | 99(0.4) | 0.019 | 79(0.4) | 95(0.4) | 0.224 |
| Chronic renal disease, n(%) | 4,255(6.6) | 2,443(10.4) | <0.001 | 2,436(11.0) | 2,128(9.6) | <0.001 |
| Cancer and metastatic solid tumor, n(%) | 1,527(2.4) | 433(1.8) | <0.001 | 819(3.7) | 404(1.8) | <0.001 |
| AIDS, n(%) | 220(0.3) | 26(0.1) | <0.001 | 43(0.2) | 26(0.1) | 0.041 |

ICD-10: International Classification of Disease 10th edition. STEMI: ST elevation myocardial infarction. COPD: Chronic obstructive pulmonary disease. AIDS: Acquired immunodeficiency syndrome.

**Supplementary Table S3**. Prevalence of conditions included in the Charlson Comorbidity Index (CCI) before and after matching by age for men and women suffering a NSTEMI

| | BEFORE MATCHING | | | AFTER MATCHING | | |
|---|---|---|---|---|---|---|
| | **Men** | **Women** | **p-value** | **Men** | **Women** | **p-value** |
| Congestive heart failure, n(%) | 7,586(16.1) | 4,892(22.9) | <0.001 | 4,238(20.6) | 4,530(22.0) | <0.001 |
| Peripheral vascular disease, n(%) | 4,447(9.4) | 1,003(4.7) | <0.001 | 2,146(10.4) | 964(4.7) | <0.001 |
| Cerebrovascular disease, n(%) | 1,860(4.0) | 966(4.5) | 0.001 | 983(4.8) | 932(4.5) | 0.233 |
| Dementia, n(%) | 539(1.1) | 708(3.3) | <0.001 | 376(1.8) | 647(3.1) | <0.001 |
| COPD, n(%) | 5,432(11.5) | 1,736(8.1) | <0.001 | 2,879(14.0) | 1,683(8.2) | <0.001 |
| Rheumatoid disease, n(%) | 451(1.0) | 511(2.4) | <0.001 | 230(1.1) | 497(2.4) | <0.001 |
| Peptic ulcer, n(%) | 203(0.4) | 70(0.3) | 0.046 | 101(0.5) | 66(0.3) | 0.007 |
| Mild and moderate/severe liver disease, n(%) | 1,303(2.8) | 415(1.9) | <0.001 | 494(2.4) | 408(2.0) | 0.004 |
| Diabetes, n(%) | 16,322(34.6) | 8,212(38.4) | <0.001 | 7,499(36.4) | 7,951(38.6) | <0.001 |
| Hemiplegia/Paraplegia, n(%) | 125(0.3) | 58(0.3) | 0.889 | 56(0.3) | 58(0.3) | 0.851 |
| Chronic renal disease, n(%) | 6,559(13.9) | 3,420(16.0) | <0.001 | 3,848(18.7) | 3,208(15.6) | <0.001 |
| Cancer and metastatic solid tumor, n(%) | 1,607(3.4) | 401(1.9) | <0.001 | 907(4.4) | 388(1.9) | <0.001 |
| AIDS, n(%) | 112(0.2) | 13(0.1) | <0.001 | 36(0.2) | 13(0.1) | 0.001 |

ICD-10: International Classification of Disease 10th edition. NSTEMI: Non-ST elevation myocardial infarction. COPD: Chronic obstructive pulmonary disease. AIDS: Acquired immunodeficiency syndrome.
